# Long-read sequencing reveals novel transcript diversity in *Polypterus senegalus*

**DOI:** 10.1080/19768354.2025.2479057

**Published:** 2025-03-31

**Authors:** Jeong Woen Shin, Bo-Mi Kim, Seung Hwan Lee, Jun Kim, Jae-Sung Rhee

**Affiliations:** a Department of Bio-AI Convergence, Chungnam National University, Daejeon, Republic of Korea; b Division of Life Sciences, Korea Polar Research Institute, Incheon, Republic of Korea; c Division of Animal & Dairy Science, Chungnam National University, Daejeon, Republic of Korea; d Department of Convergent Bioscience and Informatics, College of Bioscience and Biotechnology, Chungnam National University, Daejeon, Republic of Korea; e Graduate School of Life Sciences, College of Bioscience and Biotechnology, Chungnam National University, Daejeon, Republic of Korea; f Department of Marine Science, College of Natural Sciences, Incheon National University, Incheon, Republic of Korea; g Research Institute of Basic Sciences, Core Research Institute, Incheon National University, Incheon, Republic of Korea; h Yellow Sea Research Institute, Incheon, Republic of Korea

**Keywords:** *Polypterus senegalus*, PacBio Iso-Seq, isoforms, splicing patterns, biological traits

## Abstract

*Polypterus senegalus* is a fish species characterized by primitive traits and unique physiological-anatomical features, representing a transitional stage toward terrestrial vertebrates. Diverging from other ray-finned fishes approximately 420 million years ago, it serves as a key model for vertebrate evolutionary studies. In this study, we identified 19,295 unique *P. senegalus* transcripts using Pacific Biosciences Iso-Seq, with most classified as novel exon combinations, intron retentions, or novel start and stop codons. This high-quality, full-length transcriptome revealed previously unreported isoforms and investigated their functional roles to enhance our understanding of the genomic complexity and evolutionary traits of the species. Notably, these isoforms were identified in genes associated with *Polypterus*'s distinctive features, including limb flexibility (*ACTN3B*, *MYBPC1*), lung function (*FAM13A*, *ERBB2*, *TCF7*), and circulation (*FBN1*, *MYH11B*, *MEF2CB*). These findings offer molecular insights into early vertebrate terrestrial adaptation and the functional integration of key tissues in extant species.

## Introduction

The family Polypteridae, within the class Actinopterygii (ray-finned fishes), comprises two genera: *Polypterus* and *Erpetoichthys* (Noack et al. 1996). *Polypterus* inhabits freshwater rivers and lakes in West Africa and exhibits unique physiological traits, including aerial breathing in low-oxygen conditions and terrestrial locomotion using its pectoral fins and body (Magid et al. 1970; Lutek et al. 2022). These adaptations are believed to have diverged from those of other ray-finned fishes approximately 420 million years ago during the Devonian period (López et al. 2013). Due to its primitive characteristics, which resemble those of tetrapods, *Polypterus* serves as a valuable model for studying phenotypic innovations and the molecular mechanisms underlying tetrapod evolution (López et al. 2013).

Moreover, molecular analyses of *Polypterus* facilitate the identification of genomic regions associated with its key traits and broader genome evolution trends (Chiu et al. 2004). The chromosome-level *Polypterus* genome, published in February 2021, provides a foundation for genomic studies of this species. With a total size of approximately 3.7 Gb and a scaffold N50 of 189.7 Mb, the *Polypterus* genome demonstrates high chromosomal accuracy and completeness (Bi et al. 2021). It has been widely used to investigate the genomic characteristics and genetic mechanisms of *Polypterus*; however, its gene annotation lacks splicing-level information for this species (Inoue et al. 2003; Kato et al. 2024; Nagashima et al. 2024).

The limb flexibility of Polypterus provides valuable insights into the adaptations required for early terrestrial vertebrates to transition from aquatic to terrestrial environments, enhancing mobility in response to environmental changes (Wilhelm et al. 2015). This flexibility is attributed to simple joint structures, with the actinin alpha 3b (*ACTN3B*) and myosin-binding protein C1 (*MYBPC1*) genes implicated in the developmental differences underlying this trait.

Alpha-actinins (ACTN), which stabilize sarcomeric Z-lines by cross-linking actin microfilaments, play a critical role in muscle function. According to Christopher et al., five actin-related genes, including two *ACTN3* paralogs, exhibit muscle-specific expression (Holterhoff et al. 2009). Notably, approximately 16% of humans and birds lack *ACTN3*, highlighting significant physiological diversity in vertebrate muscle function (Holterhoff et al. 2009). Additionally, *MYBPC1* encodes sMyBP-C, a myosin-binding protein that regulates contraction and flexibility in slow skeletal muscle by stabilizing and supporting thick filaments (Geist and Kontrogianni-Konstantopoulos 2016).

The lung of Polypterus serves as a key model for studying the evolutionary transition to terrestrial vertebrates (Icardo 2018). In this context, family with sequence similarity 13 member A (*FAM13A*), erb-b2 receptor tyrosine kinase 2 (*ERBB2*), and transcription factor 7 (*TCF7*) are critical factors. *FAM13A* and *ERBB2* contribute to tissue development and lung epithelial cell repair, while *TCF7* mediates T-cell differentiation and plays a role in lung infection (Sasaki et al. 2006; Tomizawa et al. 2011; Zhu et al. 2015; Chuang et al. 2017; Eisenhut et al. 2017; Chen et al. 2023).

The circulatory system of Polypterus regulates oxygen absorption, transport, and utilization, playing a vital role in sustaining life (Capillo et al. 2021). fibrillin 1 (*FBN1*) and myosin, heavy chain 11b, smooth muscle (*MYH11B*) are essential for smooth muscle contraction and vascular wall stability. Mutations in these genes can impair contraction, leading to vascular wall degeneration and circulatory dysfunction (Kuang et al. 2012; Hemani 2023). Myocyte enhancer factor 2cb (*MEF2CB*), expressed in the late ventricular region and regulated by FGF signaling, plays a key role in heart development, performing a function similar to that of the second heart field in mice during zebrafish cardiac development (Lazic and Scott 2011; Hinits et al. 2012). Despite extensive research on these genes, their transcriptomic diversity at the splicing level remains unexplored in *Polypterus senegalus*.

A major challenge in transcriptomics at the splicing level is the comprehensive identification of isoforms across species. Earlier approaches relied on short-read sequencing, which often failed to capture critical splicing variants (Park and Chung 2019; Hu et al. 2021; Cao et al. 2022; Kim H, et al. 2022; Kim HS, et al. 2022; Yoo et al. 2022; Zhou et al. 2022; Abdellaoui et al. 2023; Lee et al. 2023; Lim et al. 2023). Recent advancements in long-read sequencing technologies have overcome this limitation by providing full-length transcript information (Byrne et al. 1786; Cho et al. 2014; Logsdon et al. 2020; Marx 2023). For instance, Pacific Biosciences (PacBio) developed the Isoform Sequencing (Iso-Seq) method, which generates high-quality, full-length transcriptome data (Gonzalez-Garay 2016). This technology has facilitated the discovery of novel alternative splicing events and isoforms associated with biological, evolutionary, and pathological traits, significantly advancing our understanding of transcriptomic diversity (Gonzalez-Garay 2016; Feng et al. 2019).

**Figure 1.**
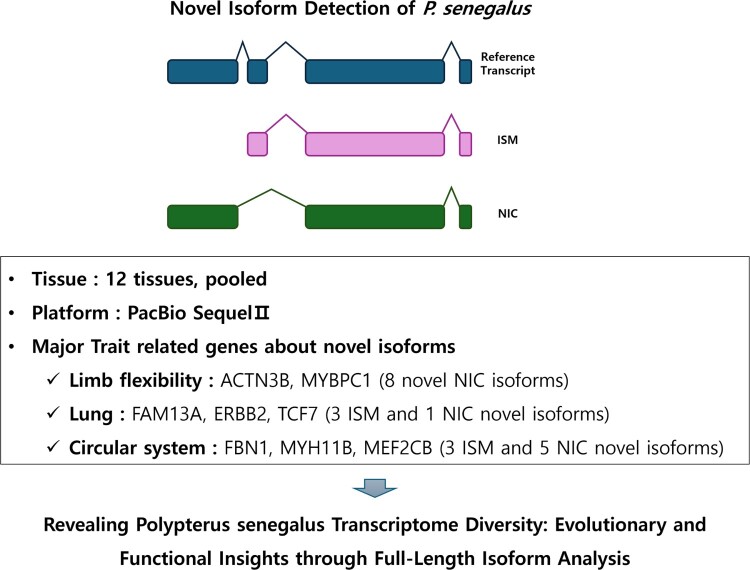
Graphical abstract. We aim to identify novel transcripts associated with major trait-related genes, including those involved in limb flexibility, lung function, and the circulatory system, in *Polypterus senegalus* using PacBio Iso-Seq data. Our ultimate goal is to elucidate the diversity of *the P. senegalus* transcriptome at the splicing level through full-length isoform analysis.

This study aimed to identify previously unreported isoforms in *Polypterus senegalus* using PacBio Iso-Seq data, thereby elucidating the genomic complexity and evolutionary traits of the species. Additionally, by structurally categorizing these novel isoforms, we aimed to identify those enriched in *P. senegalus* (Figure 1). The splicing-level transcriptome data generated in this study serve as a valuable foundation for future research in vertebrate evolution and molecular biology.

## Materials and methods

### Sample preparation and RNA sequencing

The *P. senegalus* specimen used in this study was collected by Incheon National University and processed by DNALink, Inc. (https://en.dnalink.com). RNA was extracted from 12 tissues (brain, eye, gill, ovary, kidney, liver, lung, muscle, swim bladder, gut, spleen, and skin) from the same individual. The extracted RNA samples were pooled before sequencing. Complementary DNA (cDNA) was synthesized using the SMRTer PCR cDNA Synthesis Kit and DNA Template Prep Kit 1.0. Sequencing was conducted on two cells of the PacBio Sequel II platform, and the resulting data were processed using Sequel SMRT Link 8.0 software, yielding high-quality PacBio Iso-Seq data. The read lengths of the PacBio Iso-Seq raw data were assessed using Bioawk (version 20110810): *zcat ${IsoSeq_FASTQ} | bioawk -c fastx 'OFS = ","{print length($seq)}'*. The length distribution was visualized using R (version 4.2.2) with the ggplot2 package (Wickham 2106).

### Novel transcript identification

The FASTA sequence file and gene annotation GTF file for the *P. senegalus* genome were downloaded from the NCBI database (Accession number: GCFXX). This genome assembly has a total size of 3.4 Gb and contains 29,359 genes. PacBio Iso-Seq reads were aligned to the *P. senegalus* genome using flair-align (version 2.0.0): *flair align -g ${Bi_FASTA} -r ${IsoSeq_FASTQ} --output ${PREFIX}*) (Tang et al. 2020). Errors in splice sites from the alignment results were corrected based on the gene annotation of the *Polypterus* genome using flair-correct (version 2.0.0): *flair correct -q ${PREFIX}.bed -f ${Bi_GTF} -g ${Bi_FASTA} --output ${PREFIX}*) (Tang et al. 2020). Redundant transcripts corresponding to the same isoform were collapsed to generate high-confidence isoforms using flair-collapse (version 2.0.0): *flair collapse -g ${Bi_FASTA} --gtf ${Bi_GTF} -q ${PREFIX}_all_corrected.bed -r ${IsoSeq_FASTQ} --stringent --check_splice --generate_map --annotation_reliant generate --output ${PREFIX}*) (Tang et al. 2020). Only full-length reads were included in this analysis. Full-length reads were defined as those covering at least 80% of the isoform and containing a minimum of 25 bp in both the first and last exons. To ensure splice site accuracy, reads were required to cover at least 4 bp of the 6 bp surrounding splice sites and have insertion mutations no greater than 3 bp.

### Isoform annotation and classification

The corrected and collapsed isoforms were annotated and classified by comparing them with the gene annotation of the *Polypterus* genome using SQANTI3 (version 5.2.2): *sqanti3_qc.py ${FLAIR_ISOFORM_GTF} ${Bi_GTF} ${Bi_FASTA} --force_id_ignore -o ${PREFIX} -d ${PREFIX} --report both --isoAnnotLite* (Pardo-Palacios et al. 2024). The --isoAnnotLite option was applied to facilitate a detailed evaluation of isoform structural features, splicing accuracy, and novelty.

Isoforms were categorized into eight structural classes, including full splice match (FSM), incomplete splice match (ISM), novel in catalog (NIC), and novel not in catalog (NNC). FSM isoforms were further subdivided into four subtypes based on positional differences relative to the annotated transcription termination sites. ISM isoforms were classified into five subtypes depending on fragmentation, retention, or changes in exon counts. Novel isoforms were divided into five subtypes based on whether they contained known splice junctions or entirely novel splicing sites.

### Protein structure prediction

Protein structures were predicted for novel isoforms of *P*. *senegalus* genes associated with its distinct phenotypes using AlphaFold2 and coldblood (version 1.5.5) with the following parameters: template_mode: none, msa_mode: mmseqs2_uniref_env, pair_mode: unpaired_paired, model_type: auto, num_recycles: 3, recycle_early_stop_tolerance: auto, relax_max_iterations: 200, pairing_strategy: greedy, max_msa: auto, and num_seeds: 1 (Kim et al. 2025). The per-residue local distance difference test (pLDDT) was used as a confidence score for protein structure prediction, with pLDDT values visualized using different colors in the 3D models.

## Results

### Iso-Seq revealed approximately 4500 novel isoforms of *P. senegalus*

**Figure 2.**
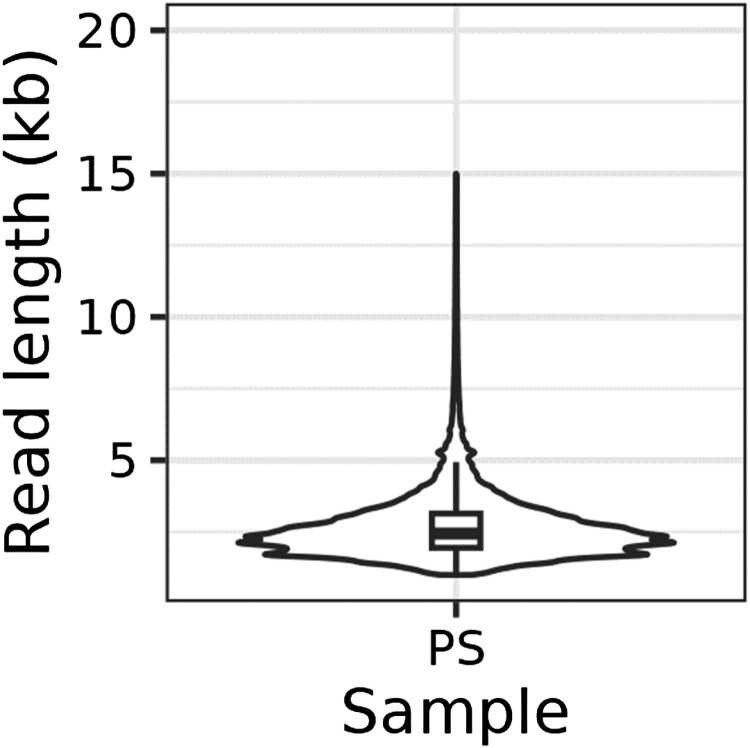
The length distribution of *P. senegalus* Iso-Seq Data. The Iso-Seq data generated for *P*. *senegalus* has an average read length of 2.74 kb, indicating its high quality and suitability for full-length trascript analysis.

We analyzed the transcriptome diversity of *P. senegalus* using RNA extracted from the following 12 tissues: brain, eye, gill, ovary, kidney, liver, lung, muscle, swim bladder, gut, spleen, and skin. Sequencing of the pooled RNA sample generated 2.85 Gb of data from a total of 1,037,557 reads (Figure 2). The average read length was 2.74 kb, ensuring high-quality data with sufficient read length and coverage.

Iso-Seq reads were mapped to the *P. senegalus* genome to correct splice sites and collapse redundant transcripts representing the same isoform. This process identified 19,295 unique transcripts, including 4547 novel isoforms. These transcripts corresponded to 13,431 genes, of which 12,106 were previously annotated, while 1325 represented newly discovered genes.

The 19,295 unique transcripts were categorized into the following classes: FSM (76.57%; known splice sites), ISM (6.40%; known splice sites but fragmented), NIC (8.30%; known splice sites but different combinations), Intergenic (3.09%), Antisense (2.81%), Genic Genomic (1.64%), Genic Intron (1.06%), and Fusion (0.13%) (Table 1). These findings support the accuracy of the previous gene annotation of the *P. senegalus* genome and validate the high quality of the Iso-Seq data, as most transcripts correspond to previously known isoforms categorized as FSM.

**Table 1.** Classification of transcripts in *P. senegalus*.

Transcript category	Isoforms count	Novel Isoforms count
FSM	14,775	27
NIC	1602	1602
ISM	1235	1235
Intergenic	596	596
Antisense	542	542
Genic Genomic	316	316
Genic Intron	204	204
Fusion	25	25
NNC	0	0

FSM: Full Slice Match; NIC: Novel In Catalog; ISM: Incomplete Splice Match; NNC: Novel Not in the Catalog.

Among the 4547 novel isoforms, NIC (35.23%) and ISM (27.16%) made up the largest proportions, with 27 additional FSM transcripts identified (Table 1). This indicates that the majority of novel isoforms consisted of either novel exon combinations with known splice sites or truncated transcripts with known splice sites.

### Splicing junction sites confirmed by Iso-Seq data

We further refined these categories to identify more specific splicing variants in *P. senegalus*. The FSM isoforms were predominantly classified as ‘Reference match’ (11,326, 76.66%), followed by ‘Alternative 3′ end’ (15.72%), ‘Alternative 3′ 5′ end’ (5.24%), and ‘Alternative 5′ end’ (1.04%) (Table 2). This indicates that the majority of FSM isoforms match the reference gene annotation, while the remaining isoforms primarily involve codon changes at the transcript ends. These differences in codon composition could indicate true alternative isoforms or misannotations of the start or stop codons in the reference gene annotation.

**Table 2.** Distribution of transcript subcategories in *P. senegalus*.

FSM		ISM		NIC	
Subcategory	Count	Subcategory	Count	Subcategory	Count
Reference Match	11,326	3′ fragment	388	Comb. of annot. junctions	596
Alternative 3′ end	2322	Internal fragment	34	Comb. of annot. splice sites	617
Alternative 3′ 5′ end	774	5′ fragment	632	Intron retention	350
Alternative 5′ end	154	Intron retention	42	Mono-exon by intron ret.	39
Mono-exon	199	Mono-exon	139		

Comb. of annot. junctions: Comination of known junctions; Comb. of annot. splice sites: Combination of known splice sites; Mono-exon by intron ret.: Mono-exon by intron retention.

The ISM isoforms were categorized as ‘5″ fragment’ (51.17%), ‘3″ fragment’ (31.42%), ‘Mono-exon’ (11.26%), ‘Intron retention’ (3.40%), and ‘Internal fragment’ (2.75%) (Table 2). This suggests that most ISM isoforms arise from the removal or misannotation of exons at either end of the transcripts.

The NIC isoforms were categorized as ‘Combination of known splice sites’ (38.51%), ‘Combination of known junctions’ (37.20%), ‘Intron retention’ (21.85%), and ‘Mono-exon by intron retention’ (2.43%) (Table 2). These findings indicate that most splicing sites annotated in the reference genome were validated by our Iso-Seq reads. Notably, no significant novel splicing junction sites were identified in the Iso-Seq data.

### The subcategories of isoforms showed similar length distributions

**Figure 3.**
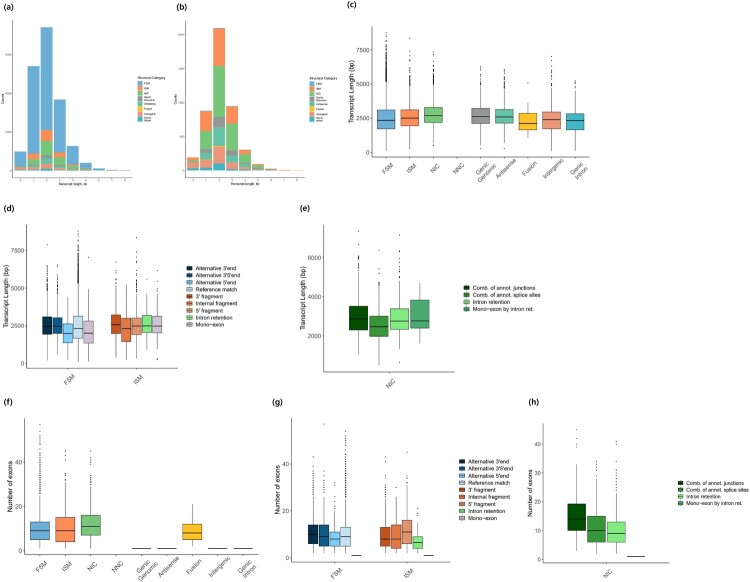
Read length and exon count distributions for total and novel transcripts in *P. senegalus*. (a) Distribution of structural categories by transcript length for the entire transcript set. The x-axis represents transcript length (kb), the y-axis represents the number of transcripts, and each color denotes a different category. (b) Distribution of structural categories by transcript length for novel transcripts. (c) Transcript length distribution across structural categories, with the x-axis representing structural categories and the y-axis representing transcript length (bp). Each category is color-coded. (d) Transcript length distribution for FSM and ISM subcategories. (e) Transcript length distribution for NIC subcategories. (f) Exon count distribution across structural classifications. (g) Exon count distribution for FSM and ISM subcategories. (h) Exon count distribution for NIC subcategories.

The average length of all 19,295 unique transcripts was 2533 bp, with a range of 83–8790 bp. Transcripts in the 2–3 kb range were the most abundant, comprising 38.4% (7416 out of 19,295), while those in the 8–9 kb range were exclusively classified as FSM or ISM (Figure 3(a)). Novel transcripts exhibited a similar distribution, with 2099 transcripts in the 2–3 kb range. Notably, one novel transcript was identified in the 8–9 kb range (Figure 3(b)). NIC transcripts had the longest average length (2811.98 bp), while Genic Intron transcripts had the shortest (2256.47 bp) (Figure 3(c)).

The subcategories within the FSM, ISM, and NIC groups showed similar length distributions. However, FSM isoforms with ‘Alternative 3′ 5′ end’ (2579.99 bp), ISM isoforms with ‘3″ fragment’ (2643.20 bp), and NIC isoforms with ‘Mono-exon by intron retention’ (3029.03 bp) were slightly longer than those in other subcategories (Figure 3(d,e)).

Most of the transcripts contained 10 or more exons. The number of exons in transcripts varied, but NIC transcripts across all main categories had the highest average number of exons (12.03 exons). Conversely, antisense, genic genomic, intergenic, and genic intron transcripts contained only one exon on average (Figure 3(f)). At the subcategory level, FSM isoforms with ‘Alternative 3′ end’ transcripts had the highest average number of exons (10.8 exons), ISM isoforms with ‘5″ fragment’ transcripts had 12.05 exons, and NIC isoforms with ‘Combination of known junctions’ transcripts had the most, with an average of 14.98 exons (Figure 3(g,h)).

### *FAM13A* and *ERBB2* exhibited novel protein sequences

Among the 4547 novel isoforms derived from 3783 genes, we focused on a small set of genes associated with Polypterus's unique biological traits, including limb flexibility, lung function, and the circulatory system. *ACTN3B* and *MYBPC1*, which are associated with limb flexibility, yielded two and six novel isoforms, respectively. *FAM13A*, *ERBB2*, and *TCF7*, known to play roles in lung development, produced two, one, and one novel isoforms, respectively. *FBN1*, *MYH11B*, and *MEF2CB*, important genes for circulatory system development, yielded one, one, and six novel isoforms, respectively (Table 3).

**Table 3.** Novel transcripts of major trait-associated genes in *P. senegalus*.

Associated trait	Gene symbol	Isoform	Chromosome	Length	Exon count	Structural category	Associated transcript	Reference length	Reference exon count
Limb flexibility	*ACTN3B*	novel3011	NC_053164.1	2752	18	NIC	novel	3001	21
		novel3012	NC_053164.1	2922	20	NIC	novel	3001	21
	*MYBPC1*	novel2186	NC_053161.1	6083	33	NIC	novel	3856	36
		novel2187	NC_053161.1	6011	32	NIC	novel	3856	36
		novel2188	NC_053161.1	5972	31	NIC	novel	3856	36
		novel2189	NC_053161.1	5966	31	NIC	novel	3856	36
		novel2190	NC_053161.1	6044	32	NIC	novel	3856	36
		novel2191	NC_053161.1	5927	30	NIC	novel	3856	36
Lung	*FAM13A*	novel1199	NC_053157.1	3313	10	ISM	XM_039750165.1	5106	25
		novel1200	NC_053157.1	3400	11	ISM	XM_039750166.1	5032	25
	*ERBB2*	novel4330	NC_053170.1	2429	16	ISM	XM_039740827.1	5478	26
	*TCF7*	novel3610	NC_053166.1	2713	11	NIC	novel	2786	12
Circular system	*FBN1*	novel3437	NC_053165.1	2665	17	ISM	XM_039771381.1	9066	59
	*MYH11B*	novel3726	NC_053166.1	6151	41	ISM	XM_039775315.1	6475	42
	*MEF2CB*	novel1861_NC	NC_053160.1	2168	1	ISM	XM_039759321.1	5301	11
		novel1979	NC_053160.1	2567	9	NIC	novel	5551	11
		novel1980	NC_053160.1	2615	10	NIC	novel	5551	11
		novel1981	NC_053160.1	2396	10	NIC	novel	5387	11
		novel1982	NC_053160.1	2348	10	NIC	novel	5387	11
		novel1983	NC_053160.1	2390	10	NIC	novel	5301	11

**Figure 4.**
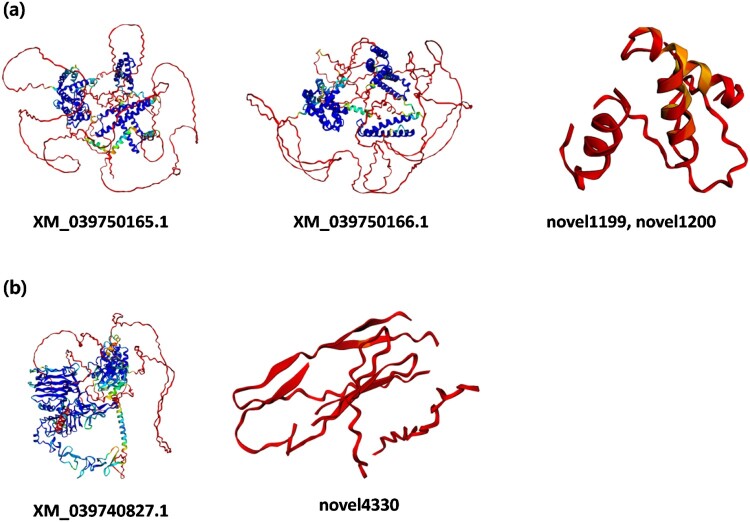
Predicted protein structures of the protein-coding transcripts associated with key traits in *P*. *senegalus*. (a) The novel isoforms novel1199 and novel1200 of *FAM13A* encode the same protein but with shorter sequences compared to the known transcripts XM_039750165.1 and XM_039750166.1. (b) The novel isoform novel4330 of *ERBB2* is significantly shorter than the known isoform XM_039740827.1.

Among these 20 novel isoforms, two novel isoforms of *FAM13A* (novel1199 and novel1200) and one novel isoform of *ERBB2* (novel4330), were confirmed as coding transcripts, and their amino acid sequences were obtained (Table 3). The two novel isoforms of *FAM13A* encoded identical protein sequences but differed in the lengths of their untranslated regions. These protein sequences were shorter than those of the canonical isoforms (XM_039750165.1 and XM_039750166.1) (Figure 4(a)). Similarly, the novel isoform of *ERBB2* had a shorter amino acid sequence compared to the canonical isoform (XM_039740827.1) (Figure 4(b)). The pLDDT scores calculated for these novel isoforms indicated low confidence in the predicted structures, with most regions scoring below 50.

## Discussion

This study used long-read RNA sequencing data to conduct a comprehensive analysis of the *P. senegalus* transcriptome at the splicing level. The Iso-Seq data, with an average read length of 2.74 kb, provided high-quality transcriptomic information, offering a robust foundation for exploring the complex gene structures and transcript diversity of Polypterus.

Iso-Seq revealed previously unreported isoforms associated with key phenotypes of *Polypterus*. Transcript structural classification (FSM, ISM, NIC, etc.) showed that FSM accounted for the largest proportion (approximately 76%), followed by ISM (6.40%) and NIC (8.30%). Among novel transcripts, the majority were distributed within NIC (35.23%) and ISM (27.16%), indicating that the *Polypterus* transcriptome exhibits newly identified, highly heterogeneous structures resulting from diverse splicing events. The discovery of novel NIC and ISM isoforms provides critical insights into previously uncharacterized splicing patterns and transcript regulation mechanisms. Additionally, the novel isoforms identified are associated with genes involved in the unique biological traits of Polypterus, including limb flexibility, the circulatory system, and lung function.

*ACTN3B* and *MYBPC1* are associated with limb flexibility (Holterhoff et al. 2009; Geist and Kontrogianni-Konstantopoulos 2016). Two novel transcripts of *ACTN3B* were identified and classified as NIC isoforms derived from combinations of known splice sites. Additionally, six novel transcripts of *MYBPC1* were discovered, all classified as NIC isoforms. These findings provide valuable insights into limb flexibility in Polypterus and the diversity of muscle function in early terrestrial adaptation. The novel NIC isoforms in *ACTN3B* and *MYBPC1* may play critical roles in regulating muscle protein structure and function, offering deeper insights into how flexibility and locomotor ability are modulated in *Polypterus* and other species. However, further validation using molecular genetics approaches is needed to confirm these findings.

*FAM13A*, *ERBB2*, and *TCF7* are associated with lung function and various pulmonary diseases in mammals (Sasaki et al. 2006; Tomizawa et al. 2011; Zhu et al. 2015; Chuang et al. 2017; Eisenhut et al. 2017; Chen et al. 2023). In this study, two ISM isoforms of *FAM13A* and one of *ERBB2* were identified, all classified as coding transcripts. These isoforms encoded shorter protein sequences than their known counterparts. Additionally, protein structure predictions revealed structural differences in these novel transcripts compared to known isoforms, highlighting the complexity of the *P*. *senegalus* transcriptome.

For *TCF7*, a novel NIC isoform was discovered. Further investigation is required to determine whether this isoform contributes to the evolutionary adaptation of respiratory structures and pulmonary function, as well as its potential relevance to disease mechanisms in modern mammals.

For *FBN1*, *MYH11B*, and *MEF2CB*, which are associated with the circulatory system, one novel ISM isoform was identified for each gene (Lazic and Scott 2011; Kuang et al. 2012; Hemani 2023). These genes play critical roles in smooth muscle contraction, vascular stability, and overall heart and circulatory system function (Lazic and Scott 2011; Hinits et al. 2012; Kuang et al. 2012; Hemani 2023). Further studies are necessary to investigate their gene expression patterns at the splicing level in Polypterus.

This study identified novel transcripts linked to the unique biological traits of *P. senegalus* using long-read RNA sequencing. These findings significantly enhance our understanding of vertebrate evolution and transcriptomic regulation, providing a comprehensive perspective on the molecular diversity and evolutionary adaptations of the species.

## Data Availability

All sequencing reads produced in this study have been submitted to the NCBI BioProject database (https://www.ncbi.nlm.nih.gov/bioproject) under accession number PRJNA1217591.
